# The Use of Carbonaceous Particle Exposure Metrics in Health Impact Calculations

**DOI:** 10.3390/ijerph13030249

**Published:** 2016-02-24

**Authors:** Henrik Olstrup, Christer Johansson, Bertil Forsberg

**Affiliations:** 1 Atmospheric Science Unit, Department of Environmental Science and Analytical Chemistry, Stockholm University, 11418 Stockholm, Sweden; christer.johansson@aces.su.se; 2 Environment and Health Administration, SLB, Box 8136, 104 20 Stockholm, Sweden; 3 Division of Occupational and Environmental Medicine, Department of Public Health and Clinical Medicine, Umeå University, 90187 Umeå, Sweden; bertil.forsberg@envmed.umu.se

**Keywords:** black smoke, elemental carbon, black carbon, urban air pollution, health effects, relative risk, PM_10_, combustion-related particles

## Abstract

Combustion-related carbonaceous particles seem to be a better indicator of adverse health effects compared to PM_2.5_ and PM_10_. Historical studies are based on black smoke (BS), but more recent studies use absorbance (Abs), black carbon (BC) or elemental carbon (EC) as exposure indicators. To estimate health risks based on BS, we review the literature regarding the relationship between Abs, BS, BC and EC. We also discuss the uncertainties associated with the comparison of relative risks (RRs) based on these conversions. EC is reported to represent a proportion between 5.2% and 27% of BS with a mean value of 12%. Correlations of different metrics at one particular site are higher than when different sites are compared. Comparing all traffic, urban and rural sites, there is no systematic site dependence, indicating that other properties of the particles or errors affect the measurements and obscure the results. It is shown that the estimated daily mortality associated with short-term levels of EC is in the same range as PM_10_, but this is highly dependent on the EC to BS relationship that is used. RRs for all-cause mortality associated with short-term exposure to PM_10_ seem to be higher at sites with higher EC concentrations, but more data are needed to verify this.

## 1. Introduction

The results from many studies indicate that short-term exposure to combustion-related carbon-containing particles is associated with more adverse health effects compared to exposure to undefined particulate matter (PM_2.5_ and PM_10_) [1,2]. In addition to having significant health impacts, the light-absorbing properties make them key players as short-lived climate forcers [3].

Black smoke (BS) has been monitored as an indicator of air-quality worldwide for decades, and has been used much longer than any other particle metrics, like PM_10_ or PM_2.5_. Measurements of BS date back to the 1920s [4], and during the London smog episode in 1952, concentrations exceeding 4000 μg·m^−3^ were reported using the BS reflectance method [5]. In 1964, the Organisation for Economic Cooperation and Development (OECD) established a standard calibration curve to convert reflectance measurements to surface weight (μg·m^−2^), which was then converted to a gravimetric concentration using the area of the black surface and sample volume through the filter [6]. Later, the mass equivalent British Standard black smoke (BSI 1969) was defined in the United Kingdom, based on domestic coal-smoke emissions, which was the main source of emissions at that time. The first WHO guideline for particulate matter was based on BS [7].

The long data records of BS have been valuable in epidemiological studies to assess associations of exposures and health effects as reviewed by Janssen *et al.* [2]. However, later, health-risk estimates were based on light absorbance (Abs), black carbon (BC) or thermo-optical methods (EC), as they are considered more specific for carbon-containing combustion particles. Several studies have shown that the different metrics (Abs, BC and EC) often are highly correlated, but the quantitative relationships between BS and BC, Abs or EC can vary widely depending on when it was performed, location, time of the year *etc.*, due to different physical and chemical characteristics that affect the optical properties of the aerosols.

In their systematic review, Janssen *et al.* [2] converted BS to EC by assuming that 1.1 μg·m^−3^ EC corresponds to 10 μg·m^−3^ BS. This is the mean-value based on simultaneous measurements at different locations in Europe and one location in the U.S. (New York). In the literature, there are several other studies with simultaneous BS, BC and EC measurements that provide other relationships between the different metrics.

In this study our aims are: (1) to review different transformations between BS and BC or EC reported in the literature. This is done to assess the degree of comparability between different epidemiological studies using the different metrics, and the possible ranges of increased mortality-risks associated with black-carbon particles; (2) To compare the estimated increase in mortality associated with short-term exposure to PM_10_ and EC (BC). We focus on short-term exposure studies, where risk estimates for either one of BS, BC or EC are reported together with risk estimates for PM_10_. The reason for the choice of short-term exposure studies is that they all use the same exposure data (urban back-ground monitors), while the few studies of long-term exposure are based on very different exposure data, which can distort the results when comparisons between these studies are performed; (3) To analyze if the effect of PM_10_ on daily mortality depends on the EC concentration.

## 2. Methods

### 2.1. Methods Addressing the Main Issues

By using the different relationships between EC and BS at various places, described in different articles, we have estimated the absolute health-risk increase for some cities with reported PM_10_ and EC (Abs, BC) concentrations. This is merely to exemplify the ranges in health risks potentially obtained using PM_10_ *vs.* EC at various locations.

Many studies have addressed the question of what is the cause of the health effects associated with PM exposure (e.g., [1,8,9]). Several authors discuss the importance of the carbonaceous particles as particularly important [8,9]. Based on the results, that the mortality risk is substantially higher at sites with high EC concentrations, we seek to further investigate if there is any relationship between the relative risks (RRs) associated with PM_10_ and the concentration of EC. This has been done by examining how well the RR associated with PM_10_ exposure correlates with the EC concentration, based on measurements from a number of cities in Europe.

### 2.2. Description of the Different Measurement Methods

In this section, we shortly describe the different measurement techniques; these are black smoke (BS), black carbon (BC), absorption (Abs) and elemental carbon (EC) that have been used in epidemiological studies. They are all intended to measure airborne carbonaceous particles, but the methods are dependent on the aerosols optical, thermal and physicochemical characteristics and the results may differ substantially [10].

#### 2.2.1. Elemental Carbon

The principle of this method is that the carbonaceous material is sampled on a quartz-fibre filter and then heated in an inert helium atmosphere, which will desorb the organic compounds, OC. The next step is based on the heating of the remaining material in an atmosphere consisting of helium and oxygen, which will combust the elemental carbon, EC. The term elemental carbon refers to the carbonaceous material that does not volatilize below a certain temperature, usually around 550 °C [11]. The heating process can, however, cause the OC compounds to pyrolyse or char, and this will cause an overestimation of EC. This can be corrected by using simultaneous measurements of reflectance (TOR) or transmittance (TOT) [2]. The TOR and the TOT method are based on the vaporization of the carbon-containing particles under different conditions, depending on temperature and oxidation. Chow *et al.* [12] found that the transmitted light is highly influenced by charring of the adsorbed organic vapors within the filter, while the reflected light is highly influenced by charring of the vapors at the surface of the filter. Compared to TOT, the EC measured by TOR is less sensitive to the temperature programs used [13].

There are no standardized guidelines for the method. Different laboratories use different temperature protocols, which will have a major impact on the results. The consequence arising from this is that the relationship between EC and the total carbon can vary by a factor up to five [14]. If the pyrolysed carbon is incorrectly accounted, it will greatly affect the calculated relationship between EC and OC [15]. Different techniques and protocols to measure EC and OC are presented in Table 1. In the German VDI method, the OC fraction is removed by chemical extraction before EC is measured by thermal desorption at 500 °C.

#### 2.2.2. Black Carbon

The term black carbon (BC) is most often referred to as the measurement of carbon particles by light absorption (transmission). The air is drawn through a filter which largely transmits light. The collected particles give rise to a blemish on the surface of the filter, and a specific absorption cross section (m^2^·g^−1^) is used to convert the absorption (m^−1^) to mass concentration (μg·m^−3^). The following equation describes the relationship [16]:

(1) $$\left[ά=\frac{A}{V}\;\text{ln}\;\frac{I0}{I}\right]$$

where ά is the absorption coefficient, A is the exposed filter area (m^2^), V is the volume of the sampled air (m^3^), I_0_ is the intensity of the incoming light before it has passed the filter, and I is the intensity of the transmitted light which has passed through the filter.

Black carbon is a mixture of EC and the light absorbing OC compounds [20]. Sometimes, it is primarily EC, as in diesel exhaust emissions, but in other emissions such as gasoline and residential wood burning, there is a more important influence of light absorbing OCs [21]. However, the influence of OC and other light-absorbing materials is also dependent on the wavelength used in the BC determination. Other light-absorbing substances, like iron oxides, may also occur in the ambient aerosols, but their contribution is normally negligible compared to EC [22]. BC is generally a good surrogate for EC, even though the correlation between EC and BC depends on the wavelength used for measuring BC. In some studies, the absorption coefficient is converted to a site specific BC mass concentration by using filter-based thermo-optical EC determination (sometimes this BC is referred to as equivalent black carbon, EBC) [23].

#### 2.2.3. Black Smoke

The black-smoke method (BS) is based on the reflection of visible light from the resulting soot blemish on a filter. The BS mass concentration is obtained by comparing the reflection to a calibration curve [6]. BS is the oldest standardized test-method for measuring soot. Originally it was developed to measure a Black Smoke Index (BSI), based on a comparison with the collected mass of PM [6]. The problem is that this conversion has remained the same, while the quantity and the chemical composition of the PM have changed significantly, especially as the coal burning in the cities has declined [4]. Consequently, black smoke can be used as an indicator of EC only at a specific time period and location, but may not reflect long-term trends in EC exposure or in emissions of EC [24]. The BS measurement method was standardized in the United Kingdom during the late 1960s through a British Standard Model (BSI, 1969). This was based on the OECD method [6]. When the black-smoke calibration was introduced, it corresponded to the total mass concentration of the PM sample. However, as mentioned, the changes in PM composition over time mean that BS does not correspond well to the total mass fraction of a sample [25]. Based on a comparison of the methods, Heal and Quincey [26] found a constant relationship between the British standard and the OECD standard (BSI_BRITISH_ = 0.85 BSI_OECD_). In the following text, the term BS refers to the OECD method.

Measuring reflectance means that there is an assumption that the light is absorbed by two passes through the filter. The interpretation of the measurement assumes that the filter and what is behind together act as a mirror. An absorption coefficient, ά, is derived according to the following equation [4].

(2) $$\left[ά=\frac{A}{2V}\;\text{ln}\;\frac{R0}{R}\right]$$

A is the exposed filter area (m^2^), V is the volume of the sampled air (m^3^), R_0_ is the intensity of the reflected light from a clean filter, and R is the intensity of the reflected light from a sampled filter. The factor 2 occurs because it is a double reflection; the light is first absorbed and then reflected through the filter [4]. However, the factor 2 may not be completely right in order to correct the double reflection; the value may vary between 2 and 2.8 depending on the type of fiber filter that is used and the sampling site [27]. The BS measurement is based on white light, while the BC measurement uses a single wavelength [4].

#### 2.2.4. Absorbance (Abs)

The term Abs has been used in many recent studies and usually refers to measurements of reflectance on a filter with sampled particles using a particle diameter cut-off of 2.5 μm (PM_2.5_). This is the same technique as for the BS measurement described above, but usually no conversion to mass concentration is performed, and for BS, there is no specific particle cut-off diameter.

### 2.3. Uncertainties Associated with the Measurement Techniques

Since all the measurement techniques mentioned above involve measurement of light absorption or light reflectance, uncertainties associated with these phenomena will somehow influence the outcomes of the measurements. All the measurement techniques involve particles extracted from air onto a filter. If not properly considered, erroneous and variable results may be recorded because the particles' properties change when they are extracted onto a filter [28].

There are a number of factors causing uncertain and variable results using the different techniques described above. The most important factors according to Fuller *et al.* [29] are:

1. Absorption and scattering depend on wavelength, and this is also dependent on particle characteristics that may vary in time and space.
2. Different shapes of the particles affect the optical properties.
3. Variable refractive index.
4. Inaccurate densities, where different types of particle agglomerates can give the same extinction effects, while the density varies. This results in uncertainties when the light extinction is translated into particulate mass.
5. Impurities, where the graphitic material is mixed with other substances causing different optical properties of the particles.

The implication for epidemiological studies is that it is difficult to compare different studies, e.g., the relative risks associated with different health outcomes. Even if studies use the same indicator, e.g., BS, the results may be affected by spatially and temporally variable optical properties of the particles, leading to uncertainties in exposure estimates. Furthermore, using fixed monitoring sites as an estimate of personal exposure is also connected with substantial uncertainties. This is particularly important for EC which shows large variability in cities (discussed more below).

### 2.4. A Summary of the Relationships between BS, BC, EC and Abs

In order to be able to compare RRs associated with exposure to carbonaceous particles from different epidemiological studies, it is necessary to use the same metric. In this section, we review published studies presenting results of comparisons of the different metrics of “soot” (BS, BC, EC and Abs). EC is here considered as the “reference” metric to which the other metrics can be related. However, it should be kept in mind that none of the metrics are absolute due to the lack of a traceable standard (as discussed above).

**Figure 1:**
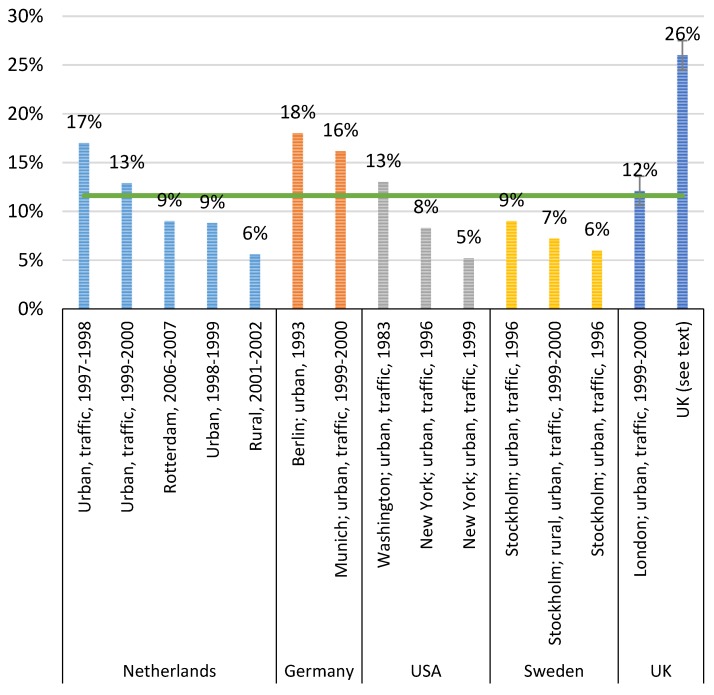
Percentage EC content in BS in the different studies. The green line corresponds to the mean value of 12%. The studies are presented in more detail in Table S1.

The results are presented in the Supplementary Materials (Table S1) and summarized in Figure 1.

A square root relationship between BC and the BS concentration was derived by Quincey [4], based on their common link to an optical absorption-coefficient, supported by a single dataset from London:

(3) $$\left[\text{BC}=\sqrt{\left(4.18\;\text{BS}+59.6\right)}-7.72\right]$$

This was later found by Quincey *et al.* [30] to be a useful approximation (within 25%) for a variety of non-kerbside monitoring sites. However, they argued that at kerbside sites, with high BS concentrations, those appear to suffer from excessive and unpredictable loading effects. Here BC is determined by aethalometry (Magee AE21 aethalometers operating at 880 nm with shadowing correction) with a specific absorbance of 16 m^2^·g^−1^.

Later, the same group (Heal and Quincey [26]) presented a general empirical relationship for transforming BSI_BRITISH_ to BC, based on measurements at five different sites across the UK. For BSI_BRITISH_ values up to 80 μg·m^−3^, the following relationship is suggested.

[BC = (0.27 ± 0.03) BSI_BRITISH_ − (4.0 ± 0.02) × 10^−4^ (BSI_BRITISH_)^2^] (4)

For BS_BRITISH_ < 20 μg·m^−3^ they recommend:

(5) $$\left[\text{BC}=\sqrt{\left(5.2\;{\text{BS}}_{\text{BRITISH}}+62\right)}-7.9\right]$$

Equation (5) is very similar to Equation (3) (Quincey *et al.* [30]).

Butterfield *et al.* [31] show that BC is highly correlated with EC at many sites in the UK, *R*^2^ > 0.80 for most of the sites and years reported. The mean EC/BC ratio for three sites during five years (2009–2013) is 0.77. Taking this value and setting BSI_BRITISH_, which is 0.85 BS_OECD_ according to Heal and Quincey [26], then EC would be in the range of 25%–28% of BS, according to Equation (4).

Schaap *et al.* [20] showed different relationships between BS and EC depending on the location in the Netherlands. Measurements in urban areas during 1998–1999 resulted in 8.8% EC content in BS, and the measurements during 2000–2001 in rural areas gave 5.6% EC content in BS. This comparison assumes that there is no time trend in the measurements.

Janssen *et al.* [32] examined the relationship between EC and the absorption, based on measurements inside and outside schools located within 400 m of motorways in the Netherlands. Absorption coefficients were converted into BS concentrations. This was done by using data on absorption of PM_10_ filters and BS concentrations, measured simultaneously at the same site. The equation obtained for the ambient measurements is:

[BS (μg·m^−3^) = 9.897 Abs − 3.663] (6)

For the EC measurements, duplicate samples were collected using Harvard PM_2.5_ impactors and Schleicher and Schuell QF 20 quartz filters and EC was found to represent a content of 17% in BS [32].

Cyrys *et al.* [33] compared EC with reflectance measurements in Sweden, Germany and the Netherlands. They used the German reference method VDI 2465 for determining EC. The measurements were performed in the period 1999–2000, and the equation obtained is:

[EC = 1.6053 Abs − 0.2620](7)

This was obtained by using the average values estimated by measurements from four rural, four urban background and four traffic sites in the Netherlands. For Sweden (Stockholm), measurements at three rural, seven urban and two traffic sites in the Stockholm area resulted in the equation:

[EC = 0.9048 Abs + 0.3560](8)

For Germany (Munich), the equation obtained is:

[EC = 2.0168 Abs − 1.1875](9)

This relationship is based on measurements from six traffic sites and six urban background sites.

The equations from Cyrys *et al.* [33] are based on the Abs measurement technique (see above). An increase of 1 unit of Abs is considered to equal an increase of 10 μg·m^−3^ in black smoke [34]. The results have also been divided by 1.25, since the VDI measurement technique is assumed to overestimate the true value of EC by 25% [15].

Hansson *et al.* [16] estimates 6.0% EC content in BS based on measurements at an urban background site in central Stockholm during 1996. The measurements of EC were performed by using a carbon analyzer (ACPM, Rupprecht and Patashnik Inc., East Greenbush, NY, USA, Series 5400), which is based on automatic sampling and thermal transformation of the carbon content to carbon dioxide. From the same study by Hansson *et al.* [16], it is also possible to derive the relationship between EC and BS by combining BC and BS measurement results. Then 9.0% EC content in BS is obtained for the same period.

Adams *et al.* [35] reported the relationship according to the following equation:

[EC = 1.35 Abs − 1.55](10)

It is based on field studies conducted in London during 1999–2000. More than 400 measurements of personal exposure of PM_2.5_ were taken for journeys by bicycles, buses, cars and underground rail-transports, where three main fixed routes were used. The persons involved in the study were carrying filters where the PM_2.5_ content was analyzed. The contribution of EC to the personal exposure of PM_2.5_ was assessed by using an optical technique and with the development and use of a size-fraction specific and site-specific calibration curve. The result was that BS had an EC content of 12.1% [35].

Measurements of BS from different stations in Berlin during the period 1989–1990 have been compared with values from OECD standard-calibration curves [19]. The values have been compared with EC, measured according to the VDI 3481 method, and the result was that BS had an EC content of 18%.

Lena *et al.* [21] measured PM_2.5_ and EC on sidewalks, and tested how the proportion of heavy traffic influenced the spatial variations in concentrations of PM_2.5_ and EC. The report is based on measurements from Hunts point, a 690-acre peninsula in the South Bronx, New York City. It is a hub in the freight transportation for the three states of New York, New Jersey, and Connecticut. Air samples were collected in the summer 1999, over a period of three weeks. The samples were collected at seven intersections, which were geographically separated. Gravimetric PM_2.5_, EC, and reflectance analysis were performed. The relationship between EC and BS is 5.2% EC content in BS.

Based on measurements on sidewalks in Harlem in New York City during a few days in July 1996, Kinney *et al.* [36] found 8.3% EC content in BS. The EC data was measured on quartz-fiber filters, which is the standard method, and the absorption coefficient was measured on PM_2.5_ filters.

Edwards *et al.* [27] determined 13% EC content in BS, based on measurements of sampled aerosols of different compositions and size distributions. Two places were used; the University of Washington campus provided a typical urban environment, while the highway tunnel U.S. 99 provided a dominating contribution of exhaust from gasoline vehicles. The relationship between the results from the British-Smoke-Shade method and the elemental-carbon method was then established, based on a mean-value obtained from all the measurements from those two places. The British black-smoke method has to be divided by 0.85 in order to achieve an OECD value according to Heal and Quincey [26]. Consequently, the content of EC in BS in their study can be estimated to be 15%.

Keuken *et al.* [37] derived 9% EC content in BS based on measurements in Rotterdam during the period 2006–2007. Parallel samples of EC and BS were collected in two-weekly periods.

The different relationships presented above are summarized in the Supplement Table S1 and graphically presented in Figure 1. As can be seen, the content of EC in BS is between 5.2% and 27%, *i.e.*, ranging by more than a factor of 5. On the other hand, the correlations between EC, BC, Abs and BS are usually very high in each study.

Most of the data come from urban environments, where the traffic is a large contributor to these particles. Comparing all studies, there is no systematic difference between the traffic and urban sites, but for individual cities (studies), EC is usually a larger fraction of BS close to traffic compared to urban background and rural sites. Neither is there a clear systematic trend in the EC fraction over time even though the earliest data are from 1983 and the most recent from 2007. The mean value of these 15 relationships is 12% EC content in BS.

## 3. Results and Discussion

### 3.1. Results

#### 3.1.1. Comparing Health Risks Associated with Short-Term Exposure to PM_10_ and EC

In Table 2, we compare increased mortality risks associated with PM_10_ and EC based on measured concentrations of PM_10_, Abs and BC at different urban sites in Europe (Putaud *et al.* [38]; Reche *et al.* [39]; Eeftens *et al.* [40]). The BC concentrations are converted to EC assuming EC = 0.77 × BC based on Butterfield *et al.* [31], as discussed above. The Abs values reported by Eeftens *et al.* [40] were converted to EC based on EC measurements at the same sites as reported by Jedynska *et al.* [41]. For PM_10_, we use the pooled estimate of 0.48% (95% CI: 0.18, 0.79) increase in all-cause mortality associated with short-term exposure per 10 μg·m^−3^. For EC, we calculate 2.5% to 13% increase in all-cause mortality per 10 μg·EC·m^−3^ when we assume 0.68% increase per 10 μg·BS·m^−3^ (95% CI: 0.31, 1.06), based on Janssen *et al.* [1] and take the minimum (5.2%) and maximum (27%) percentage EC content in BS as presented above.

**Figure 2:**
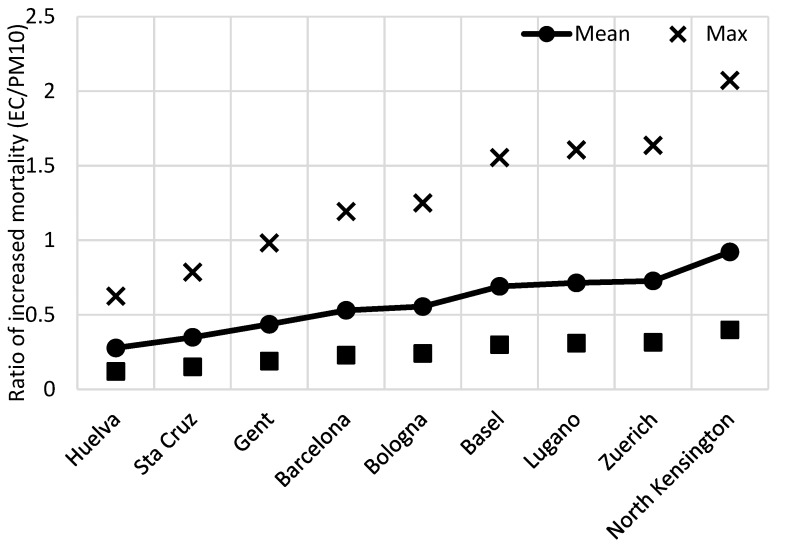
The ratio of relative increase in all-cause mortality associated with short-term exposure to measured levels of PM_10_ *vs.* exposure to our calculated concentrations of EC, derived from measured concentrations at some urban background locations in Europe. The EC content is assumed to be 77% of BC (see text).

**Figure 3:**
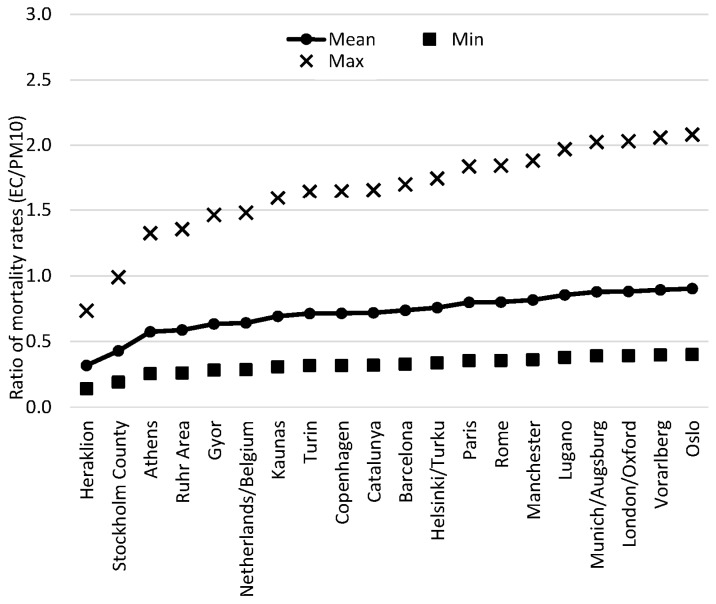
The ratio of relative increase in all-cause mortality associated with short-term exposure to measured levels of PM_10_ *vs.* exposure to our calculated concentrations of EC, derived from measured concentrations of PM_10_ and Abs at some urban background locations in Europe. The calculations are based on a 0.68% increased mortality per 10 μg·m^−3^·BS. The EC content is estimated to be 12% in BS (minimum 5.2% and maximum 27%, see text).

The ratio between mortality rates associated with EC and PM_10_ is plotted in Figure 2 and Figure 3. The ratios in Figure 2 are EC values based on BC, and in Figure 3, the EC values are obtained from the Abs measurements. As can be seen in Table 2, the mean mortality increase for PM_10_ and EC is quite similar for many of the sites. When using the reported maximum content of EC in Figure 2, estimated from the time-series studies, the increase in mortality associated with EC is up to just over two times higher compared to the increased mortality estimated from PM_10_ concentrations.

In Figure 3, we have used the same percent increase in mortality as above. The mean mortality ratio for all these sites, derived from our calculated EC concentrations to the reported mortality rates derived from a mean value of PM_10_ concentrations is 0.72, but as can be seen in the Figure 3, the range is large, from 0.32 in Heraklion to 0.90 in Oslo. Considering the maximum percentage increase in mortality estimated from the short-term exposure studies, then EC is associated with up to just over 2 times higher mortality-increase compared to PM_10_ for Oslo.

It should be noted that the concentrations used here are not actual population exposure concentrations, but the levels at one central urban background site in the cities, corresponding to the type of data used in studies of associations between daily mortality, based on daily mean concentrations. This represents a significant limitation when it comes to exposure assessment. This is especially true for EC, which shows large spatial variations within a city [11].

The ratio of PM_10_ to BC (or EC) varies in time and space depending on the relative importance of different source contributions. While EC is emitted from local combustion of fuels or biomass, PM_10_ is a bulk measure that may include more or less of non-combusted materials, such as road dust, brake or tire wear, and also secondary particulate materials (sulphate, nitrate *etc.*). PM_10_ may therefore have very different compositions and may originate from several sources depending on location and time. Exposure in close proximity to traffic have relatively higher proportion of EC, mainly due to the contribution from diesel exhaust emissions. Likewise, it is expected that sites with a large contribution from residential wood burning would have much higher increase in mortality using BC (or EC) than using PM_10_.

#### 3.1.2. Are Relative Risks of PM_10_ Exposure Dependent on the EC Concentration?

**Figure 4:**
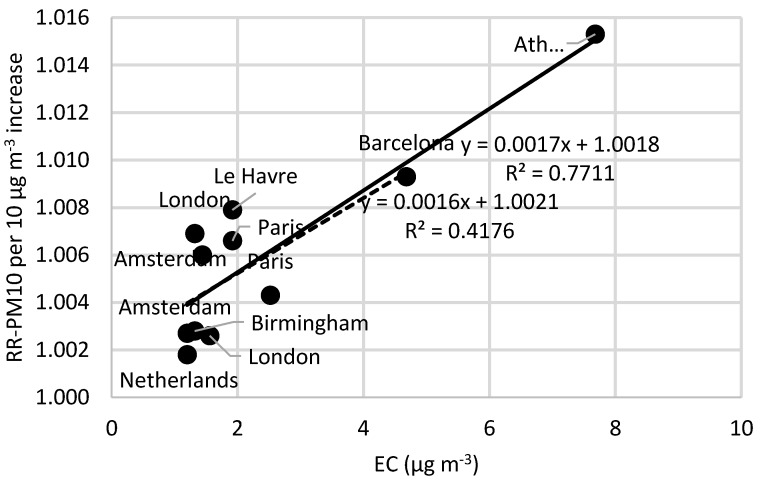
The relative risks of all-cause mortality associated with short-term exposure to PM_10_ as a function of the levels of elemental carbon (EC). The slope of the solid line is significant (95% CI). The dashed line represents the relationship where Athens is excluded. The EC concentrations are based on BS values transformed by using the calculated mean of 12% EC content in BS.

**Figure 5:**
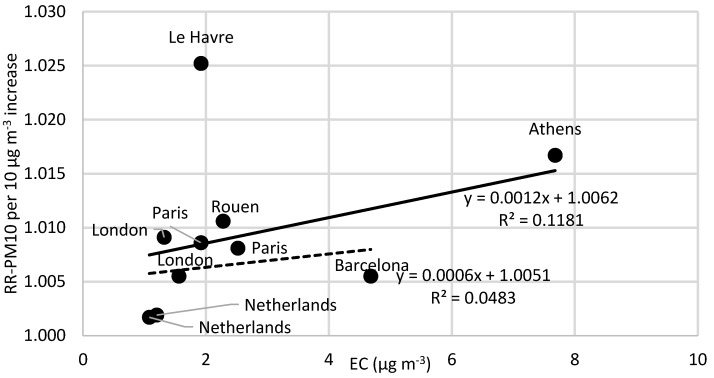
The relative risks of cardiovascular mortality associated with short-term exposure to PM_10_ as a function of the levels of elemental carbon (EC). With a 95% CI, the slope value of the solid line is not statistically significant. The dashed line represents the relationship where Athens and Le Havre are excluded. The EC concentrations are based on BS values transformed by using the calculated mean of 12% EC content in BS.

**Figure 6:**
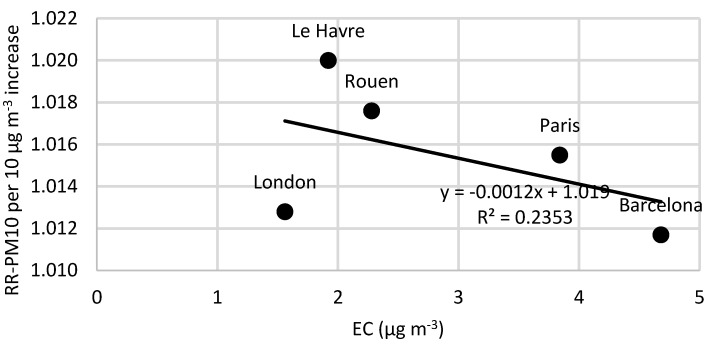
The relative risks of respiratory mortality associated with short-term exposure to PM_10_ as a function of the levels of elemental carbon (EC). The slope is not significant (95% CI). The EC concentrations are based on BS values transformed by using the calculated mean of 12% EC content in BS.

Based on the strong indications that combustion-related carbon-containing particles are more strongly associated with adverse health effects compared to exposure to PM_10_, we explore if reported relative risks (RRs) associated with PM_10_ depend on the concentration of EC. Table S2 (Supplementary Materials), presents the relative risks of short-term exposure to PM_10_ and black smoke for all-cause mortality in all ages (based on Janssen *et al.* [2]). Of the total number of 14 articles, nine of these present a larger relative risk for BS compared to PM_10_. Moreover, based on the total number of these 14 articles, nine of them show statistically significant relative risks associated with BS exposure, of which five of them also show statistically significant relative risks associated with PM_10_ exposure. Table S2 (Supplementary Materials) shows more detailed information regarding concentrations, relative risks and the studies that have statistically significant relationships. EC concentrations were estimated using the mean percentage content EC in BS (from the relations presented above). Figure 4, Figure 5 and Figure 6 show the relative risks of all-cause, cardio-vascular and respiratory mortality, respectively, associated with short-term exposure to PM_10_, plotted as a function of the EC concentrations reported in the different studies (Table S2 ). Figure 4 indicates that the relative risk of all-cause mortality associated with PM_10_ increases with increasing EC concentration. However, the correlation is highly dependent on the data from the study in Athens, and when this data point is excluded, the correlation coefficient decreases, which is shown as a separate linear regression in Figure 4. For cardiovascular and respiratory mortality (Figure 5 and Figure 6), there are less data and the slopes are not statistically different from zero (95% CI). In Figure 5, there is also a separate regression with Athens and Le Havre excluded. Athens is excluded because of the PM_10_ measurements, which are partly derived from BS, which consequently can distort the results. Le Havre is excluded because of the presence of a nickel refinery, which could potentially distort the results.

In Appendix (Figures S1–S3), we have also plotted the relative risks associated with short-term exposure to PM_10_ as a function of the concentration ratio of EC to PM_10_. This ratio may not exactly represent the true EC content in PM_10_, since if PM_10_ and EC are sampled separately with different sampling inlets, the concentrations may represent different fractions of the aerosol, e.g., PM_10_ representing more of the coarse fraction that also may contain some EC. Nevertheless, plotted in this way, the correlation (*R*^2^-value) is reduced from 0.77 to 0.58 in the case of all-cause mortality, but slightly increased from 0.12 to 0.14 for cardiovascular mortality. However, in both cases, the correlations are highly dependent on one single study (Athens).

### 3.2. Discussion

Looking at each specific site, there is usually a very high correlation between BS and EC, reflecting that they are indicators of the same source (combustion of carbonaceous material). However, when comparing all published relationships between BS and EC, there are significant differences depending on when and where the measurements have been performed. We have shown that the percent EC content in BS varies from 5% to 26%. To some extent, this is related to the varying optical properties of the aerosol and different methods used to calculate the BS concentration based on the reflectance measurements, *i.e.*, uncertainties in the BS determination. The true range in the EC content is likely much larger since the method to determine EC in different studies varies, and there is no standardized guidelines or calibration standard for determining EC. As shown by Cavalli *et al.* [14], even when using the same type of instrument (thermal-optical), different temperature protocols can cause EC concentrations to vary up to a factor of five. This means that it is not known to what degree reported relative risks for BS is due to EC, even though there is a correlation at specific sites.

If combustion-related carbon-containing particles are associated with much more adverse health effects than bulk PM_10_, one might expect to see that the RR for PM_10_ depend on the EC concentration. In fact, Figure 4 shows high correlation between the RR of all-cause mortality associated with acute exposure to PM_10_ and the EC concentration. However, the correlation is highly dependent on RR-PM_10_ from the epidemiological studies in Athens and Barcelona. Both cities occasionally have high levels of coarse (non-combustion) particles contributing to the PM_10_ concentrations. Normally, BS should not be much affected by coarse mineral-particles, but if the concentrations are very high, the light absorption by some oxides in the minerals may contribute to the absorption. Other potential reasons for the relatively weak correlation are that the EC content of PM_10_ is not a very important risk factor for mortality, or that several other factors are of varying importance depending on the site. It is also possible that the data on EC have too large uncertainties, masking the risks associated with EC exposure. Our findings that the proportion of EC does not increase the RR for PM_10_ on respiratory mortality are in line with previous observations on respiratory endpoints [42].

Table 2 shows that for most of the city sites, the mean-estimated percentage-excess mortalities are quite similar when PM_10_ and EC are used as exposure metrics in order to calculate RRs. However, even though the RRs associated with EC are about 10 times higher compared to PM_10_ per μg·m^−3^, the health impacts (excess mortality) will be similar due to the lower EC concentrations. A similar conclusion was drawn by Keuken *et al.* [37], who estimated the number of life-years saved associated with decreased long-term exposure to PM_10_ and EC in Rotterdam 1985–2008. However, as pointed out earlier by others (e.g., Janssen *et al.* [1], Grahame *et al.* [9] and Keuken *et al.* [37]), EC is a more relevant indicator than PM_10_ in order to assess the health benefits of reducing combustion-related emissions, as the health impact (reduced mortality) associated with 1 μg·m^−3^·EC exposure is about 10 times greater compared to 1 μg·m^−3^ PM_10_.

There are many possible reasons for the larger RR associated with EC exposure. Except that EC itself can be toxic, it may be a carrier of several different toxic compounds. The organic component of ambient particles is a complex mixture of many hundreds of organic compounds [11]. Polycyclic aromatic hydrocarbons are for example associated with detrimental health effects [2]. These compounds are also formed during incomplete combustion of carbon-containing fuels and thus are often highly correlated with EC.

In the urban environment, where epidemiological studies have been conducted, a large part of the emissions of EC are derived from the combustion engines in vehicles. Diesel exhaust contains a relatively large fraction of EC compared to OC, and diesel particles are also associated with a large proportion of metals. These particles are associated with metals originating from engine abrasion, lubrication oils, and from the fuels [43].

Metals has been suggested to partly explain the toxicity of EC in some studies [8]. The metals bound to particles associated with exhaust from combustion engines may also explain the increased RR-PM_10_ with increasing EC concentration. Additive effects or synergistic effects may also be important. The RR-PM_10_ for Le Havre is comparatively high, especially for cardiovascular mortality. In Le Havre, there is a nickel refinery, and exposure to nickel has proven to be particularly relevant when it comes to cardiovascular diseases [8]. However, this would assume that the nickel refinery actually leads to a relatively larger population-exposure to nickel compared to the other cities, but to our knowledge, there is no information if this is the case.

In some few studies, where BC exposure is compared to PM_2.5_ exposure, there are clear indications that BC is more harmful to the health [44]. One interesting study is Schwartz *et al.* [45], where the impact on the reduction of heart rate variability was analyzed as a result of exposure to BC and PM_2.5_. It was found that secondary PM_2.5_ was associated with reduction of heart rate variability only when it was highly correlated with BC.

Regarding the relative risks associated with EC and PM_10_, there are other uncertainties that also have to be considered. In most studies from cities, where relative risks resulting from air pollutants are estimated, they are based on the measurement values from a few measurement stations within the cities. However, in studies where geographically resolved PM levels have been compared with the measured environmental levels, the comparisons between ambient and personal exposure levels indicated only moderate correlations [46]. The difference between ambient and personal levels can be especially pronounced for EC. Since EC is combustion related, and largely derived from local sources within a city, it shows larger spatial variations within a city compared to the long-range transported aerosols, which tend to be more evenly distributed [11]. Large spatio-temporal contrasts have been seen for BC in Stockholm, reported by Krecl *et al.* [47]; for Beijing, reported by Schleicher *et al.* [48]; and for New York, reported by Wang *et al.* [49]. These results indicate that a central monitoring station may not adequately represent the short-term variability in the BC exposure of the population in the area.

The importance of accurate exposure estimates was recently demonstrated by Grahame *et al.* [44]. They analyzed associations between exposure to EC and changes in heart rate variability and compared exposure-assessments estimated from a central monitor with a more accurate method of estimating exposure. As expected, associations between EC and changes in heart rate variability were more pronounced in studies where the estimated exposures were performed with higher accuracy.

In this paper, we have only discussed effects of short-term exposure studies. As regards, studies on associations between mortality and long-term exposure to PM_10_ *vs.* EC, there are much fewer studies [1], and most of the associations (eight out of 12) are not significant, likely due to lack of statistical power. The four studies of long-term exposure and statistically significant associations with mortality are very different with respect to the exposure variables (e.g., resolution) compared to the studies of short-term exposure and daily mortality, all of which use the same type of exposure data (urban background monitors).

## 4. Conclusions

During the last years, the health risks associated with combustion-related particles have received increasing attention. The measurement methods for carbonaceous PM are important, since they may lead to large uncertainties when used in epidemiological research.

Comparing all the studies regarding the EC content in BS, there is no systematic difference between the traffic and urban sites. However, for individual cities (studies), EC is usually a larger proportion of BS close to traffic compared to urban background and rural sites. Neither is there a clear systematic trend in the EC fraction over time even though the earliest data are from 1983 and the most recent from 2007.

Transformation of BS into EC is associated with large uncertainties, due to errors in both BS and EC determinations. BS is an old measurement method based on a conversion of mass of PM that has remained the same, while the quantity and the chemical composition of PM have changed significantly over time, and for EC, there is no standardized protocol.

The few epidemiological studies with simultaneous data on BS and PM_10_ indicate that the RR-PM_10_ increases as a function of the EC concentration when it comes to times-series studies of all-cause mortality and cardiovascular mortality. However, more studies are needed to confirm this.

Estimating excess mortality in European cities by applying RRs associated with short-term exposure to PM_10_ and measured urban background concentrations gives, on average, similar effects as applying RRs for EC and corresponding urban background concentrations. Even though RR for EC is about 10 times higher than RR for PM_10_ per μg·m^−3^, the urban background concentration of EC is usually 10 times lower, making the estimated excess mortality similar. However, the ranges in estimated excess mortality for different cities are very large and associated with large uncertainties depending on, e.g., the composition of the carbonaceous aerosols. In order to reduce the uncertainty in determining the significance of combustion-related carbonaceous aerosols, a more specific and standardized methodology for measuring combustion-related particles is urgently needed.

## Figures and Tables

**Table 1:** A description of the different techniques to measure EC.

Measurement Technique	Abbreviation Means	Description	Temperture Scheme
ACPM 5400 [16]	Ambient Carbon Particle Monitor, Series 5400, from Rupprecht & Patashnick	Thermal transformation of the carbon content into carbon dioxide. Mean values of EC and OC are automatically determined *in-situ*	OC and EC are measured at 350 °C and 700 °C, respectively; Pyrolysed OC not accounted for
EUSAAR [14]	Protocol developed in the European Supersites for Atmospheric Aerosol Research project	Thermal-optical measurement protocol	Different temperature steps from 200 °C to 850 °C in a helium and an oxygen atmosphere
NOISH 5040 [14]	The National Institute for Occupational Safety and Health, Method 5040	Thermal-optical measurement method	Different temperature steps from 250 °C to 940 °C in a helium and an oxygen atmosphere
Sunset [17]	Sunset Laboratory Inc.	Thermal-optical measurement method	From 140 °C to 900 °C depending on method
VDI 2465 [18]	Verein Deutscher Ingenieure (Association of German Engineers), Method 2465	Solvent extraction of OC followed by combustion	Different temperature steps from 80 °C to 700 °C in a helium and an oxygen atmosphere
VDI 3481 [19]	Verein Deutscher Ingenieure (Association of German Engineers), Method 3481	Solvent extraction followed by combustion	Different temperature steps from 300 °C to 800 °C

**Table 2:** Measured concentrations of PM_10_ and EC, and the calculated percent increase in all-cause mortality related to short-term exposure in a number of urban background sites in Europe.

Site	Metric	Concentrations of PM_10_ and EC (μg·m^−3^)		Percent Increased Risk of All-Cause Mortality ^b^ for PM_10_ and EC			
City [Reference]	BC or Abs	PM_10_	EC ^a^	PM_10_	EC mean	EC min	EC max
Zuerich [38]	BC	24.7	1.5	1.2	0.9	0.4	2.0
Basel [38]	BC	25.7	1.5	1.2	0.8	0.4	1.9
Gent [38]	BC	37.3	1.4	1.8	0.8	0.3	1.8
Bologna [38]	BC	46.6	2.2	2.2	1.2	0.5	2.7
Barcelona [39]	BC	30.0	1.3	1.4	0.7	0.3	1.7
Lugano [39]	BC	23.0	1.4	1.1	0.8	0.3	1.8
North Kensington [39]	BC	18.0	1.5	0.9	0.8	0.4	1.9
Huelva [39]	BC	23.0	0.5	1.1	0.3	0.1	0.7
Sta Cruz [39]	BC	21.0	0.6	1.0	0.3	0.2	0.8
Heraklion [40]	Abs	38.4	1.0	1.7	0.6	0.2	1.3
Stockholm county [40]	Abs	19.1	0.7	0.9	0.4	0.2	0.9
Athens [40]	Abs	42.8	2.0	1.9	1.1	0.5	2.6
Ruhr area [40]	Abs	27.9	1.3	1.3	0.7	0.3	1.7
Gyor [40]	Abs	30.6	1.5	1.4	0.9	0.4	2.0
Netherlands/Belgium [40]	Abs	27.1	1.4	1.2	0.8	0.4	1.8
Kaunas [40]	Abs	29.5	1.6	1.3	0.9	0.4	2.1
Turin [40]	Abs	43.1	2.4	1.9	1.4	0.6	3.2
Copenhagen [40]	Abs	17.1	1.0	0.8	0.6	0.2	1.3
Catalonia [40]	Abs	35.6	2.0	1.6	1.2	0.5	2.7
Barcelona [40]	Abs	37.4	2.2	1.7	1.2	0.6	2.9
Helsinki/Turku [40]	Abs	14.8	0.9	0.7	0.5	0.2	1.2
Paris [40]	Abs	25.6	1.6	1.2	0.9	0.4	2.1
Rome [40]	Abs	37.0	2.4	1.7	1.3	0.6	3.1
Manchester [40]	Abs	17.6	1.1	0.8	0.7	0.3	1.5
Lugano [40]	Abs	23.9	1.6	1.1	0.9	0.4	2.1
Munich/Augsburg [40]	Abs	22.1	1.5	1.0	0.9	0.4	2.0
London/Oxford [40]	Abs	18.6	1.3	0.8	0.7	0.3	1.7
Vorarlberg [40]	Abs	20.6	1.4	0.9	0.8	0.4	1.9
Oslo [40]	Abs	14.8	1.1	0.7	0.6	0.3	1.4

^a^ Calculated from reported BC concentrations as 0.77*BC and from Abs as 0.812*Abs; ^b^ Assuming 0.48% increased mortality per 10 μg·m^−3^ PM_10_ and 0.68% increased mortality per 10 μg·m^−3^·BS, assuming no threshold under which no effects occur.

## Supplementary Materials

The following are available online at www.mdpi.com/www.mdpi.com/1660-4601/13/3/249/s1, Table S1. Relationships between EC and BS in different studies. Table S2. All-cause mortality in all ages related to exposure to BS and PM_10_ in different reports. (NA = not available). Figure S1. The same as Figure 4 except that the x-axis shows the proportion of EC in PM_10_ (EC/PM_10_). Figure S2. The same as Figure 5 except that the x-axis shows the proportion of EC in PM_10_ (EC/PM_10_). Figure S3. The same as Figure 6 except that the x-axis shows the proportion of EC in PM_10_ (EC/PM_10_).
