# Supplementary material for: The Use of Carbonaceous Particle Exposure Metrics in Health Impact Calculations
Source: Int J Environ Res Public Health. 2016 Feb 24;13(3):249. doi: 10.3390/ijerph13030249 (PMC4808912; doi:10.3390/ijerph13030249)
Supplement: Supplementary file 1 [file ijerph-13-00249-s001.pdf]

Supplementary Materials: The Use of Carbonaceous Particle Exposure Metrics in Health Impact Calculations

Henrik Olstrup, Christer Johansson and Bertil Forsberg

Table S1. Relationships between EC and BS in different studies.

Location	Study Period(s)	Relation	BC, EC Measurement Technique	Reference	% EC in BS
UK Birmingham, Edinburgh, Halifax, London (2 sites)	2008–2009	BC = $\sqrt{(5.2\ BS_{BRITISH} + 62)}$ − 7.9 [d]	Aethalometer (880 nm), 16.6 $m^2 \cdot g^{-1}$	[1]	25% to 27% [c]
Netherlands, within 400 m of motorways	1997–1998	BS ($\mu g \cdot m^{-3}$) = 9.897 Abs − 3.663	VDI 2465	[2]	17%
Netherlands, urban	1998/1999	EC = 0.088 BS + 0.32	Sunset	[3]	8.8%
Netherlands, rural	2001/2002	EC = 0.056 BS + 0.16	Sunset	[3]	5.6%
Netherlands; urban + traffic	1997/1998	EC = 0.17 BS [a,b]	VDI 2465	[2]	17%
Netherlands, Whole country; rural, urban, traffic	1999/2000	EC = 0.129 BS − 0.21 [a,b]	VDI 2465	[4]	12.9%
Stockholm, Sweden; rural, urban, traffic	1999/2000	EC = 0.072 BS + 0.29 [a,b]	VDI 2465	[4]	7.2%
Munich, Germany; urban + traffic	1999/2000	EC = 0.162 BS − 0.95 [b]	VDI 2465	[4]	16.2%
Stockholm, Sweden; urban, traffic	March–May 1996	EC = (0.090 ± 0.040) BS + (1.20 ± 0.35)	ACPM 5400	[5]	9.0%
Stockholm, Sweden; urban, traffic	March–May 1996	EC = (0.060 ± 0.036) BS + (1.25 ± 1.21)	ACPM 5400	[5]	6.0%
London, UK; urban + traffic	1999/2000	EC = 0.121 BS	NOISH 5040	[6]	12.1%
Berlin, Germany; urban	1989/1990	EC = 0.18 BS [b]	VDI 3481	[7]	18%
New York, US; urban + traffic	1999	EC = 0.052 BS	NOISH 5040	[8]	5.2%
New York, US; urban + traffic	1996	EC = 0.083 BS	NOISH 5040	[9]	8.3%
Washington, US; urban + traffic	Not available	EC = (0.15 ± 0.03) BS − (0.1 ± 2.4)	Thermal-optical reflectance (TOR)	[10]	15%
Rotterdam, Netherlands; Regional, Urban, Traffic	2006–2007	EC = 0.09 BS + 0.1	EUSAAR-II	[11]	9%
		Range of all			5.2%–27%
		Mean values of all relations			12%

[a] One unit of increase in Abs is considered to equal an increase of 10 $\mu g \cdot m^{-3}$·BS, according to Roorda-Knape *et al.* [12]; [b] EC concentrations measured according to the VDI method corrected by dividing all data by 1.25 as described in Schmid *et al.* [13]; [c] Assuming EC = 77% of BC (see text); [d] $BS_{BRITISH}$ = 0.85 BS, where BS = BS_{OECD}.

Table S2. All-cause mortality in all ages related to exposure to BS and PM_{10} in different reports. (NA = not available).

**Reference**	**Location**	**Relative Risk for PM10 (per 1 μg·m⁻³ Increase)**	**Relative Risk for BS (per 1 μg·m⁻³ Increase)**	**Concentration (μg·m⁻³) (Mean or Median)**		**Correlation (R) PM10 and BS**	**Period**
		95% CI (Median)	95% CI (Median)	PM_{10}	BS		
Roemer *et al.* (2001a) [14]	Amsterdam	(−1.00012)–1.00066 (1.00027)	1.00142–1.00506 (1.00324)	39	10	NA	1987–1994
Katsouyanni *et al.* (2001) [15]	Athens	1.00098–1.000208 (1.00153)	1.00041–1.00089 (1.00065)	40	64	NA	1992–1996
Katsouyanni *et al.* (2001) [15]	Barcelona	1.00058–1.00128 (1.00093)	1.00104–1.00210 (1.00157)	60	39	NA	1991–1996
Hoek *et al.* (2000) [16]	Netherlands	1.00002–1.00034 (1.00018)	1.00020–1.00060 (1.00040)	34	10	0.77	1986–1994
Verhoeff *et al.* (1996) [17]	Amsterdam	(−1.00014)–1.00134 (1.00060)	1.00020–1.00322 (1.00171)	38	12	0.51	1986–1992
Katsouyanni, (2001) [15]	Birmingham	(−1.00023)–1.00079 (1.00028)	(−1.00058)–1.00126 (1.00034)	21	11	NA	1992–1996
Katsouyanni *et al.* (2001) [15]	Cracow [a]	(−1.00056)–1.00082 (1.00013)	(−1.00062–1.00020 (−1.00021)	54	36	NA	1990–1996
Zeghnoun *et al.* (2001) [18]	Le Havre	(−1.00033)–1.00191 (1.00079)	(−1.00141)–1.00193 (1.00026)	36	16	0.70	1990-1995
Katsouyanni *et al.* (2001) [15]	London	1.00036–1.00102 (1.00069)	1.00034–1.00152 (1.00093)	25	11	NA	1992–1996
Bremner *et al.* (1999) [19]	London	(−1.00019)–1.00071 (1.00026)	0–1.00148 (1.00074)	28	13	NA	1992–1994
Zeghnoun *et al.* (2001) [20]	Paris	1.00027–1.00105 (1.00066)	1.00014–1.00072 (1.00043)	22	16	NA	1990–1995
Katsouyanni *et al.* (2001) [15]	Paris	(−1.00002)–1.00088 (1.00043)	1.00009–1.00067 (1.00038)	22	21	NA	1991–1996
Zeghnoun *et al.* (2001) [18]	Rouen	(−1.00054)–1.00102 (1.00024)	(−1.00128)–1.00198 (1.00035)	33	19	0.73	1990–1995
Anderson *et al.* (2001) [21]	West Midlands	(−1.00074)–1.00090 (1.00008)	(−1.00089)–1.00161 (1.00036)	23	13	0.64	1994–1996

[a] In this study, PM_{10} is partly derived from BS.

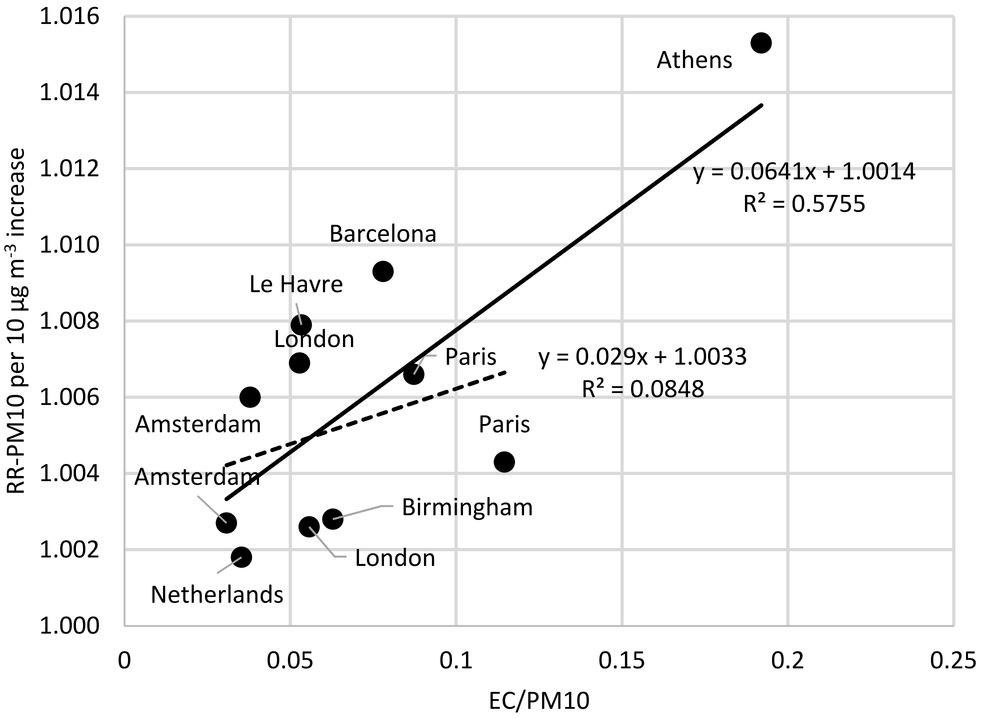

Figure S1. The same as Figure 4 except that the x-axis shows the proportion of EC in PM_{10} (EC/PM_{10}).

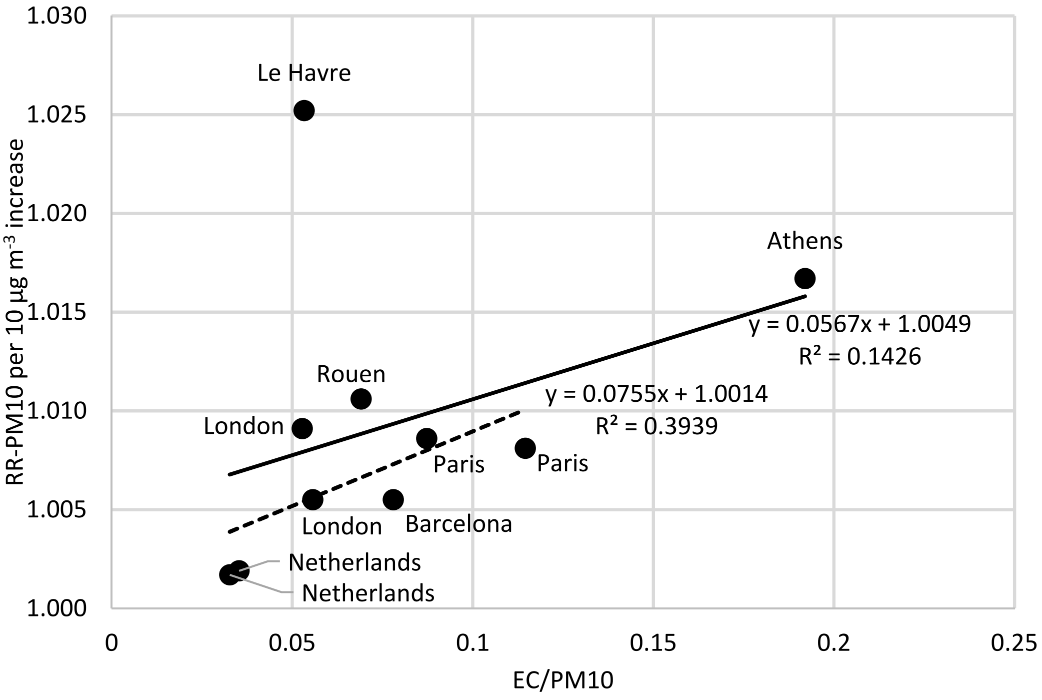

Figure S2. The same as Figure 5 except that the x-axis shows the proportion of EC in PM_{10} (EC/PM_{10}).

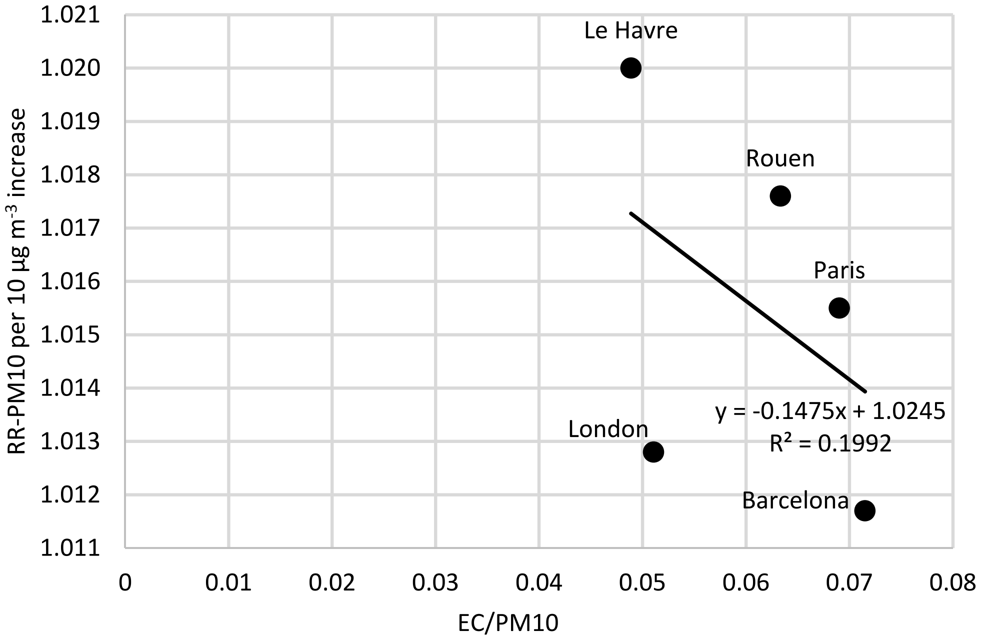

Figure S3. The same as Figure 6 except that the x-axis shows the proportion of EC in PM_{10} (EC/PM_{10}).

References

1. Heal, M.R.; Quincey, P. The relationship between black carbon concentration and black smoke: A more general approach. *Atmos. Environ*. **2012**, *54*, 538–544.
2. Janssen, N.A.H.; van Vliet, P.H.N.; Aarts, F.; Harssema, H; Brunekreef, B. Assessment of exposure to traffic-related air pollution of children attending schools near motorways. *Atmos. Environ*. **2001**, *35*, 3875–3884.
3. Schaap, M.; Denier van der Gon, H.A.C. On the variability of black smoke and carbonaceous aerosols in the Netherlands. *Atmos. Environ*. **2007**, *41*, 5908–5920.
4. Cyrys, J.; Heinrich, J.; Hoek, G.; Meliefste, K.; Lewne, M.; Gehring, U.; Bellander, T.; Fischer, P.; van Vliet, P.; Brauer, M.; *et al*. Comparison between different traffic-related particle indicators: Elemental carbon (EC), $PM_{2.5}$ mass, and absorbance. *J. Expo. Sci. Environ. Epidemiol.* **2003**, *13*, 134–143.
5. Hansson, H.C.; Nyquist, G.; Rosman, K. *Utvärdering av Sotmätningar Utförda Enligt OECD-Metoden. Resultat Från Mätningar i Stockholm Mars-Maj 1996. ITM Rapport 58*; Institutet för tillämpad miljöforskning, Stockholms Universitet: Stockholm, Sweden, 1997.
6. Adams, H.S.; Nieuwenhuijsen, M.J.; Colvile, R.N.; Older, M.J.; Kendall M. Assessment of road users' elemental carbon personal exposure levels, London, UK. *Atmos. Environ*. **2002**, *36*, 5335–5342.
7. Erdman, A.; Israel, G.; Ulrich, E. Comparative measurements of atmospheric elemental carbon using the British Black Smoke sampler and a thermal carbon analyser. *Staub* **1993**, *53*, 183–191.
8. Lena, T.S.; Ochieng, V.; Carter, M.; Holguin-Veras, J.; Kinney, P.L. Elemental Carbon and $PM_{2.5}$ levels in an urban community heavily impacted by truck traffic. *Environ. Health Perspect.* **2002**, *110*, 1009–1015.
9. Kinney, P.L.; Aggarwal, M.; Northridge, M.E.; Janssen, N.A.; Shepard, P. Airborne concentrations of $PM_{2.5}$ and diesel exhaust particles on Harlem sidewalks: A community-based pilot study. *Environ. Health Perspect*. **2000**, *108*, 213–218.
10. Edwards, J.D.; Ogren, J.A.; Weiss, R.E.; Charlson, R.J. Particulate air pollutants—A comparison of British "smoke" with optical absorption coefficient and elemental carbon concentration. *Atmos. Environ.* **1983**, *17*, 2337–2341.
11. Keuken, M.; Zandveld, P.; van den Elshout, S.; Janssen, N.A.H.; Hoek, G. Air quality and health impact of PM_{10} and EC in the city of Rotterdam, the Netherlands in 1985–2008. *Atmos. Environ*. **2011**, *45*, 5294–5301.
12. Roorda-Knape, M.C.; Janssen, N.A.H.; de Hartog, J.J.; van Vliet, P.H.N.; Harssema, H.; Brunekreef, B. Air pollution from traffic in city districts near major motorways. *Atmos. Environ.* **1998**, *32*, 1921–1930.
13. Schmid, H.; Laskus, L.; Abraham, H.J.; Baltensperger, U.; Lavanchy, V.; Bizjak, M. Results of the "carbon conference" international aerosol carbon round robin test stage 1. *Atmos. Environ*. **2001**, *35*, 2111–2121.

14. Roemer, W.H.; van Wijnen, J.H. Daily mortality and air pollution along busy streets in Amsterdam, 1987–1998. *Epidemiology* **2001**, *12*, 649–653.
15. Katsouyanni, K.; Touloumi, G.; Samoli, E.; Gryparis, A.; le Tertre, A.; Monopolis, Y.; Rossi, G.; Zmirou, D.; Ballester, F.; Boumghar, A.; *et al*. Confounding and effect modification in the short-term effects of ambient particles on total mortality: Results from 29 cities within the APHEA2 project. *Epidemiology* **2001**, *12*, 521–531.
16. Hoek, G.; Brunekreef, B.; Verhoeff, A.; van Wijnen, J.; Fischer, P. Daily mortality and air pollution in the Netherlands. *J. Air Waste Manag. Assoc.* **2000**, *50*, 1380–1389.
17. Verhoeff, A.P.; Hoek, G.; Schwartz, J.; van Wijnen, J.H. Air pollution and daily mortality in Amsterdam. *Epidemiology* **1996**, *7*, 225–230.
18. Zeghnoun, A.; Czernichow, P.; Beaudeau, P.; Hautemanière, A.; Froment, L.; le Tertre, A.; Quénel, P. Short-Term Effects of Air Pollution on Mortality in the Cities of Rouen and Le Havre, France, 1990–1995. *Arch. Environ. Health*. **2001**, *56*, 327–335.
19. Bremner, S.A.; Anderson, H.R.; Atkinson, R.W.; McMichael, A.J.; Strachan, D.P.; Bland, J.M.; Bower, J.S. Short-term associations between outdoor air pollution and mortality in London 1992–1994. *Occup. Environ. Med.* **1999**, *56*, 237–244.
20. Zeghnoun, A.; Eilstein, D.; Saviuc, P.; Filleul, L.; le Goaster, C.; Cassadou, S.; Boumghar, A.; Pascal, L.; Medina, S.; Prouvost, H.; *et al*. Surveillance des effets à court terme de la pollution atmosphérique sur la mortalité en milieu urbain. Résultats d'une étude de faisabilité dans 9 villes françaises. *Rev. Epidemiol. Santé Publ.* **2001**, *49*, 3–12.
21. Anderson, H.R; Brenmer, S.A.; Atkinson, R.W.; Harrison, R.M.; Walters, S. Particulate matter and daily mortality and hospital admissions in the West Midlands conurbation of the United Kingdom: Associations with fine and coarse particles, black smoke and sulphate. *Occup. Environ. Med*. **2001**, *58*, 504–510.

© 2016 by the authors; licensee MDPI, Basel, Switzerland. This article is an open access article distributed under the terms and conditions of the Creative Commons by Attribution (CC-BY) license (http://creativecommons.org/licenses/by/4.0/).
